# Complex and alternate consent pathways in clinical trials: methodological and ethical challenges encountered by underserved groups and a call to action

**DOI:** 10.1186/s13063-023-07159-6

**Published:** 2023-02-28

**Authors:** Amy M. Russell, Victoria Shepherd, Kerry Woolfall, Bridget Young, Katie Gillies, Anna Volkmer, Mark Jayes, Richard Huxtable, Alexander Perkins, Nurulamin M. Noor, Beverley Nickolls, Julia Wade

**Affiliations:** 1 Leeds Institute of Health Sciences, University of Leeds, Leeds, UK; 2 Centre for Trials Research, Cardiff University, 4th floor Neuadd Meirionnydd, Heath Park, Cardiff, CF14 4YS UK; 3 Department of Public Health, Policy and Systems, Institute of Population Health, University of Liverpool, Liverpool, UK; 4 Health Services Research Unit, University of Aberdeen, Aberdeen, UK; 5 Department of Psychology and Language Sciences, University College London, London, UK; 6 Department of Health Professions, Manchester Metropolitan University, Manchester, UK; 7 Centre for Ethics in Medicine, Population Health Science, Bristol Medical School, University of Bristol, Bristol, UK; 8 Department of Non-communicable Disease Epidemiology, London School of Hygiene and Tropical Medicine, London, UK; 9 Medical Research Council Clinical Trials Unit at University College London (MRC CTU at UCL), Institute of Clinical Trials and Methodology, University College London, London, UK; 10 Centre for Evaluation and Methods, Wolfson Institute of Population Health, Queen Mary University London, London, UK; 11 Population Health Science, Bristol Medical School, University of Bristol, Bristol, UK

**Keywords:** Informed consent, Clinical trials, Ethics, Underserved populations

## Abstract

**Background:**

Informed consent is considered a fundamental requirement for participation in trials, yet obtaining consent is challenging in a number of populations and settings. This may be due to participants having communication or other disabilities, their capacity to consent fluctuates or they lack capacity, or in emergency situations where their medical condition or the urgent nature of the treatment precludes seeking consent from either the participant or a representative. These challenges, and the subsequent complexity of designing and conducting trials where alternative consent pathways are required, contribute to these populations being underserved in research. Recognising and addressing these challenges is essential to support trials involving these populations and ensure that they have an equitable opportunity to participate in, and benefit from, research. Given the complex nature of these challenges, which are encountered by both adults and children, a cross-disciplinary approach is required.

**Discussion:**

A UK-wide collaboration, a sub-group of the Trial Conduct Working Group in the MRC-NIHR Trial Methodology Research Partnership, was formed to collectively address these challenges. Members are drawn from disciplines including bioethics, qualitative research, trials methodology, healthcare professions, and social sciences. This commentary draws on our collective expertise to identify key populations where particular methodological and ethical challenges around consent are encountered, articulate the specific issues arising in each population, summarise ongoing and completed research, and identify targets for future research. Key populations include people with communication or other disabilities, people whose capacity to consent fluctuates, adults who lack the capacity to consent, and adults and children in emergency and urgent care settings. Work is ongoing by the sub-group to create a database of resources, to update NIHR guidance, and to develop proposals to address identified research gaps.

**Conclusion:**

Collaboration across disciplines, sectors, organisations, and countries is essential if the ethical and methodological challenges surrounding trials involving complex and alternate consent pathways are to be addressed. Explicating these challenges, sharing resources, and identifying gaps for future research is an essential first step. We hope that doing so will serve as a call to action for others seeking ways to address the current consent-based exclusion of underserved populations from trials.

## Background

Informed consent is seen as a cornerstone in the ethical conduct of clinical trials. However, in populations or settings where there are challenges to seeking or providing consent, alternative consent arrangements may be required. These challenges may arise due to communication barriers, where a participant’s capacity to provide consent fluctuates over time, where capacity is lost during a trial, or they are deemed to lack the capacity to consent at the outset. These challenges may be particularly pronounced in emergency settings where the urgent nature of the condition and the need for immediate action preclude the ability to seek prior consent for either adults or children. Populations where consent may pose a challenge have historically been excluded from trials and are recognised as being underserved by research as a result [1]. For example, one in three patients with hip fractures have a concomitant cognitive impairment, yet eight out of ten hip fracture trials exclude this population despite evidence that those with cognitive impairment are likely to experience different outcomes [2]. Even trials in conditions associated with cognitive impairment frequently exclude people with impaired capacity to consent [3]. This exclusion of relevant subgroups of patients risks presenting biased estimates of treatment effects [4, 5] and limits the ability to provide evidence-based care for these groups.

For many of these populations, research inequity contributes to the health disparities that they already encounter [6]. For example, adults with intellectual disabilities die on average 10–15 years earlier than those without intellectual disabilities in the UK and the USA [7, 8], yet 90% of clinical trials are designed in a way that automatically excludes them from participating [9]. The importance of widening opportunities for the participation of underserved populations in research has received recognition both in the UK and beyond, resulting in national and international initiatives to improve inclusivity and diversity in the design, conduct, and reporting of clinical trials [1, 10–12]. Research funders increasingly require researchers to address issues around inclusivity and representativeness in their funding applications [13]. However, the challenges of conducting trials where consent is complex, and where consent-based exclusion denies populations the opportunity to participate in and benefit from research, have received less attention [14].

The ethical and methodological issues surrounding trials involving complex and alternative consent pathways have led to the formation of a new UK multi-institutional collaboration to collectively address some of these challenges. This collaboration forms a sub-group of the Trial Conduct Working Group in the MRC-NIHR Trial Methodology Research Partnership, consisting of members from disciplines including trials methodology, qualitative research, healthcare, bioethics, and social sciences. This paper summarises and discusses contexts where researchers may encounter particular methodological and ethical challenges around consent. The focus is on trials where the *process* of consent is challenging and alternative consent pathways are required, rather than where the *informational content* required for consent to be valid is complex [15], or where the *trial design* is complex such as a multistage randomised controlled trial [16].

Drawing on our experiences as an interdisciplinary group of researchers with an interest in complex and alternate consent pathways in trials, we will focus on key populations where consent-based challenges contribute to their exclusion: adults with communication or other disabilities [17], adults who lack the capacity to consent [18], adults whose capacity to consent fluctuates or is lost during a trial [19], and adults and children requiring emergency and urgent care [20]. The question of alternative consent pathways for children in non-emergency research will not be addressed in this article as it requires specific attention [21]. For each population, we articulate the challenges around inclusion in trials, summarise current evidence and ongoing work, and identify areas for future research. We hope that this will serve as a *cri de cœur* for others seeking ways to address the consent-based exclusion of underserved populations from trials.

## Main text

### Trials involving adults with communication, hearing, and sight disabilities

Despite the fact that the majority of legislation delineating consent processes urges professionals to make adjustments for people with communication, hearing, and visual impairments, they may be excluded from research simply due to the fact that obtaining informed consent is more challenging [22]. Communication disabilities can comprise a range of difficulties that impact a person’s ability to understand spoken or written information (sounds, words, or sentences) and express themselves verbally or non-verbally (articulate sounds/letters, select words, or use relevant grammar and sentence forms) in spoken, written, or picture form. Difficulties in accessing and comprehension of information are one of the most common barriers in consent scenarios across several diagnoses including dementia [23], stroke [24], and brain injury [25], as well as developmental disorders such as autism and learning/intellectual disabilities [26]. Other difficulties that can impede a person’s ability to access spoken or written information include hearing or visual impairments, which may or may not be associated with an underlying condition. The use of British Sign Language interpreters or translation of written materials to other languages including Braille is extremely important for those with hearing or visual impairment [27]. Beyond this, the heterogeneity amongst people with communication disabilities requires adaptations to be tailored to individual needs based on knowledge of the person’s communication strengths and difficulties. People with stroke-related language impairments (aphasia), for example, may benefit from the information being presented using active language, shorter sentences, or written keywords [28].

#### The challenges

Making changes to support communication needs is complex. Some researchers find current guidance such as the Mental Capacity Act Code of Practice [27] and Health Research Authority guidance [29] difficult to interpret and implement [30, 31]. Researchers acknowledge a lack of skills, knowledge, and confidence in being able to adapt their language and communication to meet the needs of people with communication disabilities [31]. Other barriers identified include the lack of specific training, tools, time and access to ethically approved materials [31–33].

There is limited evidence relating to the inclusion of people with communication disabilities in the informed consent process. This is in part because people with communication disabilities often have been excluded from study recruitment processes [17, 30, 31, 33–35], and because studies that have included them have tended not to report the recruitment and consent methods used [32].

#### Current research and guidance

People with communication disabilities may not be included in the informed consent process for different reasons: this group is frequently defined as ineligible for inclusion in studies per se, solely due to their communication disabilities [31]; even where included, researchers may consult proxies (e.g. family members) because they assume that people with communication disabilities lack the mental capacity to provide informed consent [17, 31, 33]; researchers may find the consent process for this group too challenging and time-consuming [31]. Reluctance to include people with communication disabilities in the consent process may follow challenges involving people with significant communication disabilities in patient and public involvement and engagement activity, and current involvement guidance does not provide specific information about how to include this group [36]. Recent UK studies have helped to contextualise these findings, by examining the legal, policy, and governance frameworks that apply to the recruitment of people with communication disabilities [30, 37, 38]. Whilst not specific to trials, these frameworks provide guidance for facilitating the inclusion of this group in the informed consent process. This includes recommendations to co-produce information materials with people with communication disabilities and to adapt communication environments and processes to improve their accessibility. These recommendations are supported by research that has developed and tested communication methods to support decision-making during the informed consent process for people with post-stroke aphasia [22, 32, 39] and intellectual disability [33, 40].

In recent examples, researchers have been able to create and use accessible consent materials and implement these within stroke trials [41–43] using practical, evidence-based resources [22, 44, 45]. These have been co-produced to ensure the language is accessible, readable, and accompanied by transparent visual representations and alternative mediums (video for example). Furthermore, the recent ASSENT [46] and CONSULT [47] projects have developed inclusive consent guidance and resources to aid researchers.

#### Future research

More research is required to explore the inclusion of people with communication disabilities in the informed consent process in trials, in terms of current practice and professional and participant experience. Most existing research appears to have focused on two main groups: people with post-stroke aphasia and people with intellectual disabilities. Future research should explore the experiences and needs of people with different types of communication disabilities, for example, people living with dementia or with other progressive neurological conditions.

Further research is required to develop and evaluate additional tools, resources, and training interventions to support researchers to work with people with communication disabilities more easily and effectively during the informed consent process [37]. Evaluation should include the exploration of usability, acceptability to professionals and participants, and cost-effectiveness. In addition, studies should explore how researchers can form successful and equitable collaborations with people with communication disabilities as part of trial public involvement and engagement activity in order to co-produce inclusive consent processes and materials [48].

### Trials involving adults whose capacity fluctuates or is lost during a trial

Informed consent can only be obtained from individuals who have the capacity to give consent. Fluctuating capacity can refer to situations where a person’s condition is cyclical (moving from an acute phase to a recovery phase) [49] or where their capacity is influenced by other factors including but not limited to health or environment [50, 51]. It can also relate to capacity that is task-specific, where an individual may have the capacity to consent to certain aspects of a trial but may struggle to give informed consent to all aspects or understand long-term follow-up processes.

Fluctuating capacity raises three main challenges: (1) the potential exclusion of those believed to have fluctuating capacity where no clear assessment process is in place, (2) the need for a process of consent-taking at each data collection time point, and (3) the need to incorporate planning for a loss of capacity, temporary or otherwise, when creating trial processes, patient information and consent materials. Without forward planning, unanticipated lost capacity during data collection may lead to withdrawal and/or missing data and the unnecessary exclusion of participants [52].

#### The challenges

Capacity is often framed (and commonly understood and implemented by recruiting staff) in binary terms as something a person has or does not have [53, 54], which has been critiqued in certain populations and cultural contexts [55]. In England and Wales, the Mental Capacity Act 2005 makes it clear that capacity is task-specific. Once assessed, capacity is not an end point but an ongoing process of engagement with a participant.

An intention to carry out capacity assessments is often alluded to in trial protocols without further detail being given on why certain individuals will be assessed, who will conduct assessments, and what criteria they will use [9]. The Mental Capacity Act 2005 and the Code of Practice (2007) exist to protect individuals, but not to impede their right to participate in research, something researchers should acknowledge. However, there is a lack of practical guidance in these documents which results in uncertainty about how researchers should best assess capacity. This can lead to inconsistent approaches to assessment. Capacity should be assumed in individuals, and capacity assessments should also only take place after the individual has been given clear information, appropriate to their needs, and there is a question raised about their ability to provide informed consent. This again raises challenges for trials where standard information is required that can be complex, lengthy, and difficult to adapt to the needs of different groups (for example, people with communication disabilities) [56, 57].

#### Current research and guidance

Suggestions for alternative forms of consent that may support those whose capacity fluctuates have been developed by researchers working with specific populations [58], including process consent in dementia research [59]. These distinguish between time and task-specific capacity and the capacity to take a longitudinal view, implying an understanding of future risks and benefits [49]. However, research to date often focuses on distinct populations, e.g. people receiving palliative care [60], people living with dementia, and stroke survivors. Attention to managing fluctuations in capacity is less often seen in population-wide trials. To reduce blanket exclusions for certain populations, and misuse of lack of capacity being used as an exclusion criterion, further research resulting in clear guidance is required.

Standardised tools for capacity assessment have been developed, but there is no gold standard for the assessment of capacity in clinic or in research, nor is there an agreement that any one tool can sufficiently capture the complexity of capacity assessment [61]. Current Mental Capacity Act-compliant tools remain difficult to adapt to the heterogeneity of the populations for whom capacity fluctuates [62–64]. Capacity assessment processes are also often only employed in certain trials which anticipate that their target population will require them.

#### Future research

Consent needs to be understood as task- and time-specific and requiring accessible information. Research is needed to generate guidance on what to do if capacity is lost during follow up and it must be based on a defined process of establishing the wishes of participants at the initial consent stages. More evidence is required on the best methods for capacity assessment and how to support researchers to assess capacity. Trials need to build protocols for how to prevent exclusion of those who may fluctuate in capacity to consent and on how to manage data collection from those whose capacity does fluctuate.

### Trials involving adults who lack the capacity to consent

Even with support, some people will be unable to provide their own consent to take part in a trial. The exclusion of adults who lack the capacity to consent has been widely documented [18, 65, 66] and is due to a range of intersecting methodological and systemic barriers to their inclusion [34]. Specific consent-based challenges include the complexity of the patchwork of legal frameworks that govern trials involving adults lacking capacity both within the UK [67] and internationally [68], and the uncertainties of applying them in practice [69]. In the UK, clinical trials of an investigational medicinal product involving adults lacking capacity are governed by the Medicines for Human Use (Clinical Trials) Regulations [70], with other types of trials covered by mental capacity legislation such as the Mental Capacity Act in England and Wales [71]. In both cases, there are provisions for an alternative decision-maker to be involved in enrolment decisions, usually a family member or close friend, or someone acting in a professional capacity who is not involved in the research if no one is able or willing to act in a personal capacity [70, 71]. For clinical trials, the alternative decision-maker is termed a legal representative and provides consent based on the person’s presumed will [70], and for other types of research, they act as a consultee and are asked to provide advice about participation based on the person’s wishes and preferences [29]. However, little guidance is available to families and health and social care professionals about their role in making decisions about trial participation, nor the legal basis for their decision [72].

#### The challenges

Due in part to this legal complexity, a lack of knowledge about research involving adults who lack capacity, and paternalistic attitudes generally, may result in gatekeeping practices by researchers and health and social care professionals towards this population [73, 74]. Involving health and social care professionals as a consultee or legal representative relies on them having the time and willingness to be involved. Some may be concerned about being unable to determine or represent the wishes and preferences that a person may hold and so may decline to become involved [38]. Other challenges arise due to the difficulties in identifying and contacting consultees and legal representatives [75]. Even when they have been identified, family members are less likely to agree to research participation on the person’s behalf than patients themselves [76]. This may be due to families’ difficulties in knowing what that person’s wishes and preferences would be about participation [77]. People rarely discuss their research preferences in the event that they might lose capacity, and there is no current mechanism in the UK for prospectively appointing a consultee or legal representative to make decisions about research [78].

Procedures for identifying and approaching consultees and legal representatives are one of the issues that research ethics committees (RECs) consider when reviewing applications for trials involving adults who lack capacity, alongside arrangements for assessing capacity to consent where required [29]. However, RECs’ resistance to the inclusion of adults who lack capacity in a trial, and whether there is sufficient justification to do so, is cited as one of the greatest barriers to their inclusion [79, 80]. RECs do not interpret the legal frameworks consistently or, at times, correctly, with inaccurate terminology and requirements being cited [37, 72, 81]. There have been calls for greater explicitness and accuracy when applications for ethical review of these studies are both submitted and reviewed [72, 81] and for incorporating more adaptations and accommodations into the recruitment process such as ensuring information is cognitively accessible [37].

#### Current research and guidance

Recent research has identified a number of barriers and facilitators to involving adults lacking consent in trials [19, 34] leading to the creation of guidance, for example, for recruiting adults with impaired mental capacity at the end of life in research [19]. Recent initiatives to address the inclusion of underserved groups in research more broadly, such as the NIHR INCLUDE project [1], have led to the development of the INCLUDE Impaired Capacity to Consent Framework which is a tool to help researchers to design and conduct trials that are more inclusive of people with impaired capacity to consent [82].

Other studies have focused on the role of personal consultees and legal representatives. This includes a study that found that making ethically complex decisions about research on behalf of someone else can be challenging for many family members, with some experiencing a decisional and emotional burden as a result [83]. Current work includes the development of the first decision aid for families making decisions about research on behalf of someone who lacks the capacity to consent [84] which is currently being evaluated as a ‘Study Within a Trial’ (or ‘SWAT’) (CONSULT) [85] and the development of resources to help researchers [47].

#### Future research

Despite the ongoing work, there is a need for a more sustained effort to ensure that these groups have an equitable opportunity to participate in trials. More research is needed into how researchers can design more inclusive trials, and the involvement of health and social care professionals as nominated consultees, and the use of professional legal representatives when necessary. Unlike questions about why other underserved groups have been excluded from research, the legal position regarding people who lack capacity is that their *inclusion* requires justification [29]. Clearer guidance is required on how this justification is understood and interpreted.

A number of recommendations for further research at a policy and legislation level have been previously made, including proposals by the Nuffield Council on Bioethics [86] that consideration be given to extend the role of the welfare attorney in England and Wales to include decisions about research, both within the Mental Capacity Act [71] and the Clinical Trials Regulations [70]. There is also uncertainty about the role of Lasting Power of Attorney in decisions about research participation [78], with families wanting greater support and guidance when making decisions [83].

### Adult and paediatric emergency and urgent care trials

Trials involving adults in emergency situations may encounter additional complexities. The challenges of obtaining consent from patients who are suddenly unable to communicate or convey their own wishes are encountered in trial contexts ranging from intrapartum [87] and acute coronary syndrome [88] to acute stroke where it has been described as the rate-limiting step in treatment RCTs [89]. Emergency and urgent care trials are conducted in a range of settings including prehospital [90] and critical care [91].

Historically, children have not received evidence-based healthcare in emergency and critical care settings due to their exclusion from trials arising from similar practical and ethical issues to those encountered in adult trials in these time-critical settings [92]. In order to increase the chances of saving a child’s life, treatments need to be given without delay, so there is no time to seek informed consent from parents or legal representatives. Even if there is a brief window of opportunity for recruitment discussions, parents may not be present or may be highly distressed and lack the capacity to make an informed decision about the use of their child’s information and potential ongoing involvement [93].

#### The challenges

Emergency research is when treatment needs to be given urgently [94] and recruitment cannot be delayed until the patient either regains capacity or a consultee or legal representative can be found [95]. In such circumstances, research without prior consent (RWPC, also referred to as ‘deferred consent’) is permissible in many jurisdictions including the USA, Canada, parts of Australasia, and the UK through both the Mental Capacity Act [71] and the 2006 Amendment to the 2004 EU Clinical Trials Regulations [96]. However, there are variations in the provisions for RWPC in emergency research, both between and within countries [97]. Within the UK, for example, the law in Scotland does not provide any ‘exemptions’ or alternatives for the involvement of adults not able to consent for themselves in clinical trials in emergency situations [94]. This meant that trials such as RECOVERY-RS [98], which compared respiratory strategies for patients with COVID-19 respiratory failure, could not recruit Scottish patients. Similarly, the UK-REBOA trial in life-threatening torso haemorrhage was unable to recruit in Scotland despite being coordinated from there [99].

In recognition of the need to conduct these vital trials with children, various legal frameworks for paediatric trials have also been amended nationally and internationally, enabling research to be conducted without prior consent. In 2008, UK legislation was amended to allow research without prior consent in such circumstances [100], yet there was a lack of knowledge about how and when research teams should broach these research discussions with parents in a way that avoided further burdening families. There was also a need for guidance to inform what should happen when a child dies after trial enrolment without parents’ prior knowledge or consent. Despite the 2008 legislation that enabled much-needed research on emergency treatments for children, there was hesitancy amongst clinical and research communities about conducting trials involving critically ill children [101].

The use of RWPC in both adult and child populations is ethically complex, with diverse views about the acceptability of enrolling acutely ill patients without consent [102]. There are particular challenges around gaining ethical approval for the use of RWPC in borderline or ‘middle ground’ cases where a patient may be conscious or coherent, yet their condition or the lack of time limits the possibility of informed consent [103]. These, and other challenges [34], can lead to consent-based recruitment bias which means that patients enrolled in RCTs may not necessarily be representative of critically ill patients in clinical practice [20, 104]. This has the potential to cause harm by obscuring any treatment effect [105].

#### Current research and guidance

A recent study in the UK (Perspectives Study) explored consent and recruitment in adult critical care research [106] and identified strategies to enhance consent and recruitment processes. This led to the development of good practice guidance and other resources including an accessible animation for members of the public [107]. An animation aimed at adults enrolled in emergency care research which describes RWPC was developed by another research team (CoMMiTED Study) [108]. Systematic reviews have explored stakeholders’ views about the acceptability of RWPC [109], including ethnic minority populations’ views [110]. Such studies have found that RWPC is generally acceptable to patients, families, and practitioners but highlighted the importance of contextual factors.

The CATheter infections in Children Trial (CATCH) was the first UK trial to include research without prior consent when comparing the effectiveness of different types of central venous catheters to prevent bloodstream infections in children. An embedded study (called CONNECT [111]) explored parent and practitioner views and experiences of recruitment and consent and found that parents were momentarily shocked or surprised when they were informed that their child had already been entered into CATCH without their consent [101]. However, initial concerns were often quickly addressed by practitioner explanations about why it had not been possible to seek consent before enrolment and how the trial interventions were already used in clinical care. To prevent burden and assist decision-making, parents stated it was important for the research staff to assess the appropriate timing of research discussions after a child’s enrolment in a trial. They suggested that the researcher should consult with the bedside nurse about appropriate timing and only approach parents after the initial emergency situation has passed, when a child’s condition has stabilised [101]. The CONNECT study used these findings alongside wider research, involving practitioners, families [112], and children [113] with experience in emergency care, to develop guidance for future paediatric and neonatal trials [114]. Since its publication in 2015, CONNECT guidance has informed the successful conduct of five studies. This includes the first clinical trial of a drug for long-lasting seizures (EcLiPSE trial), which successfully recruited to time and target with a 93% consent rate and led to changes in clinical guidelines for children in status epilepticus [115].

#### Future research

Research into consent in emergency settings is high on the trials methodological research agenda and was identified as a research priority by Clinical Trials Units in a UK survey [116]. Areas for future research involving adults identified by the Perspectives Study included the need for evidence-based guidance on the procedures for professionals acting as a consultee or legal representative and identifying strategies to communicate with relatives of critically ill patients about research, including where a participant enrolled without prior consent subsequently dies [106]. The NIHR RfPB-funded study ‘ENHANCE’ will begin in 2023 and aims to address this gap in knowledge through the involvement of bereaved families and other key stakeholders.

Ongoing work in paediatric populations aims to assess and refine CONNECT guidance in low- and middle-income countries. Further work is needed to explore views on research without prior consent in underserved populations, such as parents who do not speak English and who are often excluded from qualitative studies and guidance development.

The need for more guidance for RECs who are reviewing emergency and urgent care trials and support for consent processes for patients and members of the public who join research teams and advise on studies, has also been highlighted [106, 109, 117].

## Conclusions

The need for alternative consent processes that address the inadvertent exclusion of certain populations has been detailed in this article. Drives for trial efficiency, lack of funding, or time for adaptation often result in the exclusion of certain populations. However, inequities in health outcomes will continue to be exacerbated by health research until trials become more inclusive of underserved populations. Alongside methodological innovation, further research is required to establish good practice, develop evidence-based guidance, and support skill acquisition in the global research workforce. Our key recommendations for future research are summarised in Table 1. Importantly, this should be done in collaboration with people with lived experience and those who care for them.

**Table 1:** Summary of key recommendations for future research to address methodological and ethical challenges surrounding trials involving complex and alternate consent pathways

Underserved group from trials involving complex and alternate consent pathways	Key recommendations for future research
Adults with communication, hearing, and sight disabilities	Explore the current practice regarding the inclusion/exclusion of people with communication disabilities in the informed consent process in trials, including exploring professional and participant views and experiences across a range of populations and settings.
	Explore the experiences and support the needs of people with a range of different types of communication disabilities (e.g. people living with dementia or with other progressive neurological conditions) in order to enhance their opportunity to participate in trials.
	Develop and evaluate additional tools, resources, and training interventions to support researchers to work with people with communication disabilities more easily and effectively during the informed consent process. Evaluation should include an exploration of usability, acceptability to professionals and participants, and cost-effectiveness.
	Explore how researchers can form successful and equitable collaborations with people with communication disabilities as part of public and involvement activities in order to co-produce inclusive consent processes and materials for trials.
Adults with fluctuating capacity or where capacity is lost during a trial	Develop guidance for researchers on the actions and processes required if participants who provide consent subsequently lose capacity during follow-up. This should include the process for establishing the wishes of participants during the initial consent stages and the requirement for providing accessible information to support decision-making at all stages.
	Generate better evidence on appropriate methods to assess the capacity to consent to trials and explore how best to support researchers to assess capacity where indicated.
	Establish how to support researchers to design trials that are inclusive of adults whose capacity to consent may fluctuate during a trial, including through built-in protocols on how to manage consent processes and data collection procedures for those whose capacity does fluctuate.
Adults lacking the capacity to consent	Explore how the justification for the inclusion of adults who lack capacity is understood and interpreted by researchers and research ethics committees (RECs) and develop clear guidance for researchers and RECs to help aid consistency in ethical review processes and outcomes
	Explore the role and experiences of health and social care professionals acting as nominated consultees and professional legal representatives in emergency and non-emergency trials and establish their informational and support needs. Develop guidance for health and social care professionals acting as consultees/legal representatives about their role and the legal basis for their decision.
	Explore stakeholders’ views about extending the role of the welfare attorney in England and Wales to include decisions about research under the Mental Capacity Act and explore the wider policy and legislative implications.
	Explore stakeholders’ views about the role of Lasting Power of Attorney (LPA) in decisions about research participation and explore relatives’ informational and support needs when making decisions about research when holding LPA.
Adults and children requiring emergency care	Identify strategies to communicate with relatives of critically ill patients about research, including in situations where a participant enrolled without prior consent subsequently dies.
	Explore underserved populations’ views about research without prior consent where there are additional intersecting factors, for example, parents of critically ill children that are not proficient in the English language or who have additional language or communication needs.
	Develop guidance on consent processes in emergency and urgent care trials for REC members undertaking the ethical review and for patients and members of the public who join research teams and advise on emergency and urgent care trials.

The populations detailed above are not the only areas where consent is complex or alternative pathways are required. Some trials have complex consent processes, not because of their recruited population, but due to an innovative treatment or trial design, such as cluster RCTs and Trials within Cohorts (TwiCs) [118]. As we progress with the innovation of trial design, we must progress methodological innovation in consent at the same pace or risk leaving certain populations behind. Many of the methodological lessons learnt and proposed adjustments, such as the routine provision of accessible information, could also benefit other underserved groups including those with lower literacy levels and English language proficiency, as well as the wider population of potential research participants.

The TMRP Complex and Alternate Consent Pathways group is driving forward this research agenda in the UK and is open to new members to share methodological learning. We have updated the NIHR Clinical Trials Toolkit [119] to reflect the most up-to-date research in this area. However, as this commentary has shown, current guidance remains limited in its utility and requires greater clarity and practical applicability for researchers, participants, family members, and ethical review committees. We are keen to use the momentum of the group to identify others with an interest in this area in order to collaboratively develop the research agenda and address the consent-based ethical and methodological challenges in trials. Many of these issues are not restricted to the UK but are encountered internationally, which raises additional challenges when conducting multi-national trials [58, 97, 120]. We encourage researchers from other regions and jurisdictions to share their experiences and ongoing research programmes and to contribute to developing an international research agenda to address these global challenges.

## Data Availability

Not applicable as no dataset was generated.
